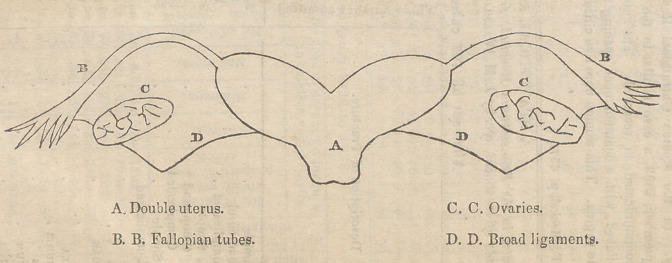# An Account of a Double Uterus

**Published:** 1838-01-17

**Authors:** P. B. Goddard

**Affiliations:** Prosector in the University of Pennsylvania


*An Account of a Double Uterus, by* P. B. GODDARD, *M. D. Prosector in the University of Pennsylvania; with a drawing.*

A few weeks since, whilst Prof. Horner was engaged in his demonstrations, his attention was arrested by something peculiar in the pelvis of a female subject, supposed to be about 50 years of age. On examination it proved to be a *double uterus*, and it was retained for the Anatomical Museum.

The neck of the uterus at the lower part presents the usual appearance, and there is nothing remarkable in the *os tincæ*; but at the upper part, the neck bifurcates to the right and to the left, each division leading to a cavity, distinct in body and fundus.

The cavity in each body is not regularly triangular, as is usual, but it is of an oblongated ovoidal shape. The *rugæ*, commonly found in the neck, and termed *arbor vitæ* by anatomists, are in this case exclusively confined to the common cervix, and do not appear to extend into the two divisions.

The external shape of each body, is an irregular ovoid, without any disposition to triangularity, and the size of each is about two-thirds the usual dimensions of the uterus in a healthy state. The appearance of the preparation is pretty well represented by the accompanying outline.

A. Double uterus. B. B. Fallopian tubes.

C. C. Ovaries. D. D. Broad ligaments.

Appended to the fundus of each division of this uterus, there is a single round and broad ligament; with a single ovum, and fallopian tube. The ovaries are of the usual size, and covered with cicatrices.

Nothing certain can be learned of the history of the patient.

As it is the intention of Prof. Horner to give himself a detailed account of this preparation at some future period, this brief notice may suffice for the present.

Philadelphia, January 6th, 1838.

Vicious conformations of the gestative organ, are perhaps not so uncommon as is generally supposed. Tiedemann and Gmelin are inclined to attribute them to an arrest in development, and a persistence of the primitive formation—primitive deviations of formation consisting in an excess of the formative power, being but rarely known.* As the frequency of its occurrence is involved in the important subject of super-fœtation it becomes an object of interesting inquiry. In the Medico Chirurgical Review for September, 1823, a case similar to the one just communicated is recorded. The uterus was bilobed, with a common neck opening into a single vagina. This woman bore five children. Her first delivery was tedious and painful, the succeeding three easy; she died of peritoneal inflammation in the last, which was laborious. The last child had occupied the right lobe. Several other instances we shall mention, but many more are on record. Lobstein mentions a case of two distinct uteri; Saviard and Duverney dissected a female who had two wombs, the one opening into the vagina, the other into the rectum, Vallesneri mentions an exactly similar case. Maygrier (*vide* Midwifery,) met in his rooms with a uterus bicornis, and gives a drawing of it. Eisenmann, Hunkelmœller,† Morgagni and Dupuytren all record instances; also there is one mentioned in the Leipsic Commentaries. Teidemann‡ relates an example of double uterus with a double cervix; and M. West, in a paper read before the Academy of Medicine, mentions a similar case. Martin and Thamm have noticed all these irregularities at length. [Eds.]

* J. F. Meckel, p. 457.

† De vaginâ et utero duplici, Berlin 1818.

‡ Observation d'une grossesse chez une femme dont la matrice etait double. *Journal Compl. des Sciences. Sc, Méd. vol.* vi, *p.* 371. Also, Mad. Boivins, *art. des accouchmens.* p. 85.

## Figures and Tables

**Figure f1:**